# Unilateral medial frontal cortex lesions cause a cognitive decision-making deficit in rats

**DOI:** 10.1111/ejn.12751

**Published:** 2014-10-27

**Authors:** Paula L Croxson, Mark E Walton, Erie D Boorman, Matthew F S Rushworth, David M Bannerman

**Affiliations:** 1 Friedman Brain Institute, Icahn School of Medicine at Mount Sinai1470 Madison Avenue, New York, NY, 10029, USA; 2 Department of Experimental Psychology, University of OxfordSouth Parks Road, Oxford, OX1 3UD, UK; 3 Wellcome Trust Centre for Neuroimaging, University College LondonLondon, UK

**Keywords:** decision, lesion, neglect, rat, T-maze

## Abstract

The medial frontal cortex (MFC) is critical for cost–benefit decision-making. Generally, cognitive and reward-based behaviour in rodents is not thought to be lateralised within the brain. In this study, however, we demonstrate that rats with unilateral MFC lesions show a profound change in decision-making on an effort-based decision-making task. Furthermore, unilateral MFC lesions have a greater effect when the rat has to choose to put in more effort for a higher reward when it is on the contralateral side of space to the lesion. Importantly, this could not be explained by motor impairments as these animals did not show a turning bias in separate experiments. In contrast, rats with unilateral dopaminergic midbrain lesions did exhibit a motoric turning bias, but were unimpaired on the effort-based decision-making task. This rare example of a cognitive deficit caused by a unilateral cortical lesion in the rat brain indicates that the MFC may have a specialised and lateralised role in evaluating the costs and benefits of actions directed to specific spatial locations.

## Introduction

Neural circuits comprising the medial frontal cortex (MFC), the core subregion of the nucleus accumbens (NAc) and midbrain dopaminergic projections from the ventral tegmental area (VTA) are important in evaluating how much effort to expend for reward (Floresco *et al*., 2008; Botvinick *et al*., 2009; Hillman & Bilkey, 2010). For example, in a T-maze choice task, MFC-lesioned rats are less inclined to choose the goal arm in which they have to climb a barrier for a larger reward and are more inclined to choose the low effort/low reward (LR) option (Walton *et al*., 2002). Rather than causing cost aversion *per se*, lesions and/or inactivations of the MFC (and more specifically of the dorsal anterior cingulate cortex) impair rats' ability to overcome a particular type of cost, namely effort, to gain a greater benefit when there is a smaller, more easily obtained reward also available (Walton *et al*., 2003; Schweimer & Hauber, 2005; Rudebeck *et al*., 2006; Floresco & Ghods-Sharifi, 2007). Notably, a similar finding has been observed in the T-maze choice task after disrupting dopamine (DA) transmission systemically or by targeted dopaminergic lesions in NAc (Salamone & Correa, 2012).

Given the similar effects of disrupting either MFC or DA in the NAc, there has been increasing interest in how these regions may interact when making effort-based choices. In one study, Hauber & Sommer (2009) showed that disconnection of the NAc and anterior cingulate cortex, using an asymmetrical (contralateral), excitotoxic lesion approach, which disrupts communication between structures in both hemispheres, impaired effort-based decision-making on the T-maze barrier task. In contrast, lesions of the same target structures in the same hemisphere did not disrupt behavioural performance. The disconnection lesion approach makes use of the fact that cognitive operations in rodents and non-human primates are not lateralised to the degree that they are in humans, and therefore can continue to be supported by communication between intact brain structures in one hemisphere (Parkinson *et al*., 2000; Gaffan & Wilson, 2008).

However, it is not clear whether interaction between the MFC with the mesolimbic dopaminergic system is also required to regulate effort-based decision-making. We therefore investigated this question in a pilot study by making excitotoxic MFC lesions in one hemisphere (MFC lesion) and depleting DA by injecting 6-hydroxydopamine (6-OHDA) into either the dopaminergic midbrain (midbrain–DA lesion) or the NAc (NAc–DA lesion), in either the same or opposite hemispheres, and tested rats on the T-maze barrier task (Salamone *et al*., 1994; Rudebeck *et al*., 2006). Surprisingly, we found that, not only were the contralateral-lesioned rats significantly impaired compared with sham controls, but, unexpectedly, the ipsilateral groups were also impaired, suggesting that unilateral lesions of one of the target structures alone affected the ability to make decisions about how much effort to exert for a reward.

Therefore, in a second experiment, we compared the performance of rats with unilateral lesions of either the MFC, NAc–DA or midbrain–DA on effort-based decision-making, and contrasted the results against performance on two tests of motoric turning bias. The results provide a rare example of a selective cognitive decision-making deficit that is separate from motor function, arising from unilateral damage in a higher-order association cortical structure, and indicate a biased lateralisation of function in the rodent MFC.

## Materials and methods

### Subjects

Male Lister Hooded rats (Harlan, OLAC, UK) weighing 250–300 g at the start of testing were kept on a 12-h light–dark cycle (lights on at 07:00 h) and maintained at 85% of their free-feeding weight. All procedures were carried out in accordance with the UK Animals Scientific Procedures Act (1986) and were approved by the UK Home Office, and experiments were carried out in accordance with the EU Directive 2010/63/EU on the protection of animals used for scientific purposes.

### Apparatus

Training and testing on the cost–benefit decision-making task in Experiments 1 and 2 were carried out in an enclosed, high-sided T-maze, placed 80 cm above floor level. The T-maze consisted of a start arm and two goal arms, each 60 cm long and 10 cm wide, surrounded by 40-cm-high walls, and painted a uniform grey color throughout (Walton *et al*., 2002). For the effort component, wedge-shaped wire mesh barriers (with a range of vertical heights from 15 to 30 cm) were placed at the midpoint of the appropriate goal arm. Rats scaled the vertical side and then descended the slope (hypotenuse) to reach the food pellets. For each rat, one arm of the T-maze was designated throughout as the high reward (HR) arm, the other as the low reward (LR) arm. The HR arm always contained four pellets and the LR arm contained two pellets (45 mg food-reinforcement pellets – Formula A/I; P.J. Noyes, Lancaster, NH, USA). The left–right allocation of the HR arm was counterbalanced across lesion groups.

### Habituation and pre-operative training

Details of training and testing procedures are provided in Walton *et al*. (2002). Briefly, after habituation to the maze, rats received 3 days of ‘forced’ trials (two trials per day), in order to expose them to the reward contingencies in each goal arm (HR or LR). They were constrained to explore only one goal arm of the maze on each trial. On each day rats received an equal number of forced trials to the right and left goal arms. The sequence of left and right arm visits was arranged pseudorandomly, and there were no more than two consecutive trials to the same goal arm.

Subsequently, rats were given reward size discrimination (choice) trials in which they were allowed a free choice of either goal arm (HR vs. LR). No barriers were present during this stage. Having visited an arm, the rats were removed from the maze before they could enter the other, non-visited arm. On each day, each rat initially received two forced trials, one to each goal arm, followed by 10 choice trials, with an inter-trial interval (ITI) of approximately 5 min. This protocol was used throughout all experiments.

After the rats had learned the reward size discrimination, they then underwent barrier training. A 15-cm-high wire mesh barrier was introduced into the HR arm. When each rat had achieved an average score of 90% HR choices with the 15-cm barrier, the barrier height was increased to 20 cm. Rats were given three training days with a 20-cm barrier and 3 days with a 25-cm barrier. Finally, they received 1 day of preliminary training with a 30-cm barrier in the HR arm before pre-surgical baseline testing began.

### Pre-surgery testing (Experiments 1 and 2)

All rats were then tested for 3 days prior to surgery (Block A). The LR arm contained two pellets and no barrier, whereas the HR arm had four pellets but the rats were required to scale a 30-cm barrier in order to access the larger reward. Rats received 10 trials per day.

### Surgery

Rats were anaesthetised with isoflurane, and placed in a stereotaxic frame with the head level between bregma and lambda. An incision was made along the midline, and a section of bone overlying the injection sites was removed. Intracerebral injections were made using a 5-μL syringe with a specially adapted 34-gauge needle mounted on the stereotaxic frame. All rats received injections of 20 mg/mL desipramine in sterile water (1 mL/kg i.p.), 30 min before the first intracerebral injection, in order to aid the survival of non-dopaminergic, monoaminergic neurons. DA depletion in either the dopaminergic midbrain (midbrain–DA) or NAc (NAc–DA) was produced by injecting 6-OHDA at 4 mg/mL in an ascorbic acid solution (1 mg/mL in 0.9% saline). Excitotoxic MFC lesions were made with quinolinic acid [0.09 m in phosphate-buffered saline (PBS)]. For details of injection sites and volumes, see Table 1. Infusions were made manually with a volume of 0.1 μL injected over 30 s, followed by a 30-s wait time to allow the toxin to diffuse away from the injection site. This was repeated until the appropriate volume had been injected. After the final infusion at a given injection site the needle was left in place for an additional 3 min. The incision was then sutured and the rats were allowed to recover for at least 14 days with unlimited access to food.

**Table 1:** Stereotaxic coordinates and injection volumes of toxins for each lesion type, for rats in both Experiment 1 and Experiment 2

Lesion	Toxin	Volume (μL)	AP	ML	DV
MFC	Quinolinic acid (0.09 m in PBS)	0.5	+3.0	± 0.8	−3.0
		0.5	+3.0	± 0.8	−1.5
		0.5	+2.3	± 0.8	−2.0
		0.5	+1.6	± 0.8	−2.0
		0.5	+0.9	± 0.8	−2.0
		0.5	+0.2	± 0.8	−2.0
NAc–DA	6-OHDA (4 mg/mL in 1 mg/mL ascorbic acid)	0.4	+1.1	+1.9	−6.5
		0.4	+1.6	+1.8	−6.6
		0.4	+2.2	+1.2	−5.9
Midbrain–DA	6-OHDA (4 mg/mL in 1 mg/mL ascorbic acid)	0.2	−5.5	+0.5	−7.8
		0.2	−5.9	+0.5	−7.7
		0.2	−6.3	+0.5	−7.6

6-OHDA, 6-hydroxydopamine; AP, anterior-posterior; DA, dopamine; DV, dorsal-ventral; MFC, medial frontal cortex; ML, medial-lateral; NAc, nucleus accumbens core; PBS, phosphate-buffered saline.

For Experiment 1, rats were divided into five groups (see Table 2 for group sizes): (i) rats with crossed MFC and midbrain–DA lesions in which lesions were made in contralateral hemispheres (midbrain–DA–X); (ii) ipsilateral MFC and midbrain–DA lesions, with both lesions in the same hemisphere (midbrain–DA–I); (iii) crossed MFC and NAc–DA lesions (NAc–DA–X); (iv) ipsilateral MFC and NAc–DA lesions (NAc–DA–I); and (v) sham operated controls. Midbrain–DA lesions will deplete DA throughout the striatum (dorsal and ventral) and across the entire forebrain, but should not affect glutamatergic or γ-aminobutyric acid-ergic neurons in midbrain structures. NAc–DA lesions will deplete DA from the NAc but, importantly, spare cell bodies in this structure. Excitotoxic MFC lesions will destroy all cell bodies in this cortical region, thus disrupting the unilateral glutamatergic projections from MFC to the NAc (but sparing other glutamatergic projections to the NAc from other cortical/subcortical structures). The major connections between the MFC and the mesolimbic dopaminergic system are within-hemisphere (Phillipson, 1979; Sesack *et al*., 1989). Thus, the crossed lesion approach leaves one of each structure intact in opposite hemispheres, but they are disconnected, and so the circuitry on both sides of the brain is disrupted (e.g. Parkinson *et al*., 2000). The ipsilateral control groups received two unilateral lesions in the same hemisphere. These animals also have one of each structure intact, but they remain connected in the intact hemisphere.

**Table 2:** Rats in Experiment 1 were given combinations of unilateral lesions (an excitotoxic cell body lesion in MFC or a 6-OHDA dopaminergic lesion of either the midbrain or NAc); rats in Experiment 2 received single unilateral lesions; the shams in each experiment were different rats

	Group	Lesion	*n*
Experiment 1	Midbrain–DA–X	Left midbrain–DA + right MFC	5
	NAc–DA–X	Left NAc–DA + right MFC	5
	Midbrain–DA–I	Left midbrain–DA + left MFC	5
	NAc–DA–I	Left NAc–DA + left MFC	6
	Sham	–	7
Experiment 2	MFC	Right MFC	4
		Left MFC	5
	Midbrain–DA	Left midbrain–DA	9
	NAc–DA	Left NAc–DA	7
	Sham	–	9

DA, dopamine; MFC, medial frontal cortex; NAc, nucleus accumbens core.

Assignment to groups was counterbalanced according to both pre-operative performance levels (% choices to the HR arm), and left–right allocation of the HR arm. Half of the MFC lesions were made in the left hemisphere and half in the right hemisphere. All midbrain–DA and NAc–DA lesions were made in the left hemisphere. Thus, half of the lesions were ipsilateral controls and half were contralateral disconnection lesions. Rats undergoing sham surgery experienced the identical surgical procedure to the lesioned rats except that they did not receive intracerebral injections.

For Experiment 2, the rats were divided into four groups: (i) unilateral MFC; (ii) unilateral NAc–DA; (iii) unilateral midbrain–DA; and (iv) sham operated controls. Allocation to groups was counterbalanced as above. To match the previous experiment, approximately half of the MFC lesions were made in the left hemisphere, and half in the right hemisphere. Again, all NAc–DA and midbrain–DA lesions were made in the left hemisphere. The groups are shown in Table 2.

### Post-surgery testing – Experiment 1

Following post-surgical recovery, rats were re-tested on the maze with a 30-cm barrier in the HR arm and no barrier in the LR arm for 6 days (Block B). As during training, each rat was given two forced trials (one each to the HR and LR arms), followed by 10 choice trials on each day of testing. The rats then received 7 days of testing with a 20-cm barrier in the HR arm (data not shown). Finally, they were then tested for a further 3 days with an identical 20-cm barrier in both the HR and LR goal arms (Block C). This block of testing is a control designed to equate the effort across the two choices so that the decision no longer requires cost–benefit integration. Instead it tests whether the rats are able to: (i) discriminate between differently sized rewards; (ii) to associate the different reward magnitudes with the different goal arms of the maze; while (iii) also controlling for their ability to make the effortful response (i.e. climb barriers).

### Post-surgery testing – Experiment 2

The rats in Experiment 2 underwent the same testing procedure on the T-maze barrier task as those in Experiment 1, except that in Block C they were tested for a total of 5 days with an equal-sized barrier in each goal arm (rather than 3 days as in Experiment 1). Although after 3 days of training with the double-barrier condition, unilateral MFC rats in Experiment 2 had shifted their choice behaviour back to choosing the HR arm on the majority of trials, they were still performing below the levels of the other groups and so an additional 2 days of testing were undertaken to determine whether these rats could attain equivalent performance levels to controls on the reward size discrimination. Furthermore, in order to assess whether animals in Experiment 2 had an asymmetric, postural and/or motoric deficit caused by the unilateral lesions, we also used two standard tests of rotational (turning) behaviour.

#### 45° grid test

A 10 × 10 cm wire mesh grid with 1 cm^2^ spacing was suspended from a shelf, 1 m above the ground, sloping downwards at a 45° angle (Schallert *et al*., 1982). The rat was placed on top of the grid, facing downwards. Rats will quickly turn around through 180° so as to face upwards, before climbing back towards the safety of the shelf. Each time the animal turned through 180° in order to face upwards, the direction in which it turned was recorded (clockwise or anticlockwise). Each rat received 10 trials with an ITI of approximately 5 min.

#### Rotational behaviour after administration of d-amphetamine (AMPH)

Rotational behaviour was also assessed under the influence of AMPH in transparent, circular bowls (30 cm in diameter; Hawkins & Greenfield, 1992). Four rats were tested simultaneously under low-lighting conditions. A video camera mounted on the ceiling was used to record activity. For the first 15-min period (‘Pre’), a baseline measure of rotational activity was established in the absence of any drug treatment. The rats were then removed from the bowls, and each animal was given an i.p. injection of AMPH (1.5 mg/kg, dissolved in 0.9% saline at 1.5 mg/mL). The numbers of 360° rotations (clockwise and anticlockwise) were then recorded for 30 min post-drug injection. Data were analysed as two 15-min post-injection periods (‘Post1’ and ‘Post2’).

### Histology

Rats were anaesthetised with sodium pentobarbital (200 mg/kg) prior to transcardial perfusion with physiological saline for 3 min and 4% (wt/vol) paraformaldehyde in 0.1 m phosphate buffer for 10 min. The brains were removed and kept in 4% paraformaldehyde solution at 4 °C for at least 2 days before being transferred to cryoprotectant (30% wt/vol sucrose phosphate) for 2 days. Coronal sections (50 μm, 2 : 4) were cut with a base-sledge microtome. Half of the sections were stained immunohistochemically *in situ* with a monoclonal antibody for tyrosine hydroxylase (TH; Immunostar, Hudson, WI, USA; catalogue no. 22941; 1 : 4000 concentration), to check for DA-reactive terminals in both the NAc–DA and midbrain–DA lesion groups. Briefly, sections were washed and incubated for two nights in primary antibody and blocking serum (1% foetal calf serum, 5% normal goat serum and 0.2% Triton in PBS). Sections were then quenched with hydrogen peroxide and incubated for 1.5 h in secondary antibody (goat anti-mouse peroxidase-conjugated antibody; Sigma-Aldrich, Dorset, UK) and then developed with diaminobenzidine before being mounted, dehydrated and coverslipped. The rest of the sections were stained with Cresyl violet to assess the extent of the excitotoxic MFC lesions. Lesions are described in the nomenclature used by Paxinos & Watson (1998).

## Results

### Histology

There were no systematic differences in size or placement of the lesions across Experiments 1 and 2. All rats that were lesioned were included in the behavioural analyses (i.e. no animals were excluded on histological grounds).

#### MFC lesions

**Fig 1:**
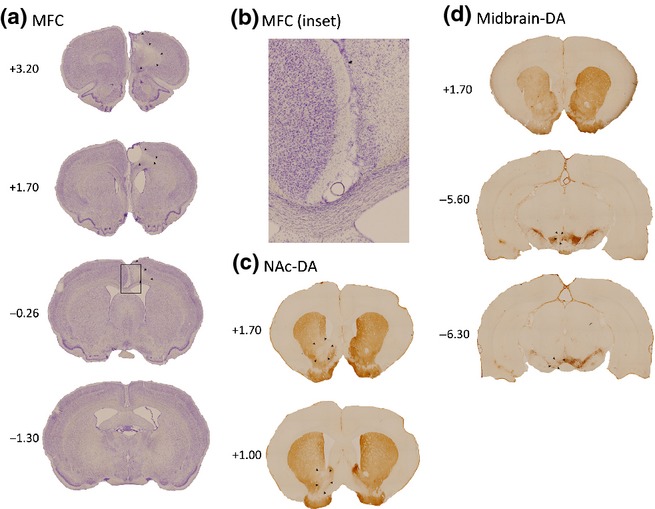
Photomicrographs of coronal sections taken from a representative unilateral lesioned brain for (a) medial frontal cortex (MFC) lesions (a lesion in the right hemisphere is shown here), (b) Inset - close-up view of the MFC lesion from 0.26 from Bregma, location shown by the black box in (a). The most minimal damage was in the unlesioned (left) hemisphere, even close to the midline, (c) nucleus accumbens core (NAc) dopamine (DA) lesions (NAc-DA; left hemisphere), (d) dopaminergic-midbrain lesions (midbrain-DA; left hemisphere), showing DA depletion in the striatum in the top panel, and in the dopaminergic midbrain in the bottom two panels. The extent of each lesion is indicated with black arrows. The numbers indicate anterior/posterior extent with respect to Bregma.

MFC lesions included the prelimbic and infralimbic cortex, as well as the Cg1 and Cg2 regions of the anterior cingulate cortex. They were complete cortical lesions and did not extend subcortically (Fig.1a). They extended ventrally to include the prelimbic cortex and infralimbic cortex. The anterior cingulate cortex lesion was complete in all but seven out of 30 cases, where there was sparing of the posterior part of Cg1 and Cg2 at caudal sections around Bregma. In six out of 30 cases the lesion extended slightly into M2, but this was not extensive and most of M2 was left intact. All the MFC lesions were unilateral apart from some minimal damage to the most superficial layer of cells in the cortex of the unlesioned hemisphere at the midline in six out of 30 cases (see inset, Fig.1b). These lesions were highly comparable to those reported in the study by Walton *et al*. (2002).

#### NAc–DA lesions

DA depletions in the NAc were generally restricted to the core subregion, and core damage was complete in all cases (Fig.1b). There was a limited amount of damage to the shell region in 10 out of 14 cases and to the caudate-putamen in six out of 14 cases.

#### Midbrain–DA lesions

Damage to the dopaminergic midbrain was unilateral, and generally complete, except for some sparing of the most anterior portion in five out of 18 cases (Fig.1c). Although the co-ordinates for these lesions were aimed at the VTA, the damage extended laterally into posterior regions, causing consistent, partial DA depletion in the medial substantia nigra in all cases. Consistent with DA cell loss in both the VTA and substantia nigra, there was reduced TH staining in both the NAc and caudate-putamen, an example of which is shown in Fig.1c (top panel). Thus, these lesions were effectively more extensive than the NAc–DA lesions.

### Behavioural analysis – Experiment 1

**Fig 2:**
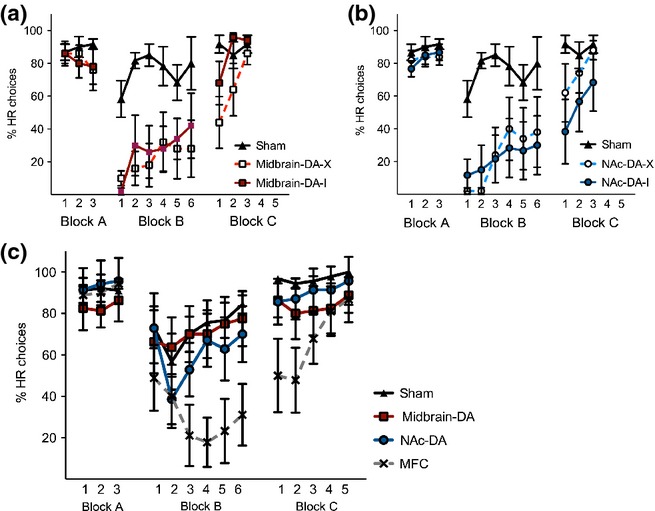
Graphs showing the mean performance of rats with crossed (NAc–DA–X) or ipsilateral (NAc–DA–I) lesions (a), or crossed (midbrain–DA–X) or ipsilateral (midbrain–DA–I) lesions (b) in Experiment 1 in each of the experimental blocks. Block A – pre-operative test with barrier in the high reward (HR) arm; Block B – post-operative test with barrier in the HR arm; Block C – barriers in both arms. Shams are the same in both (a) and (b); repeated in each graph for clarity. (c) Graph showing the mean performance of rats with sham or unilateral lesions (MFC, NAc–DA or midbrain–DA) in Experiment 2 in each of the blocks. Error bars are standard error of the mean. Note – in (a) Block C, the error bars are present, but very small. Midbrain–DA, midbrain dopamine lesions; MFC, medial frontal cortex excitotoxic lesions; NAc–DA, nucleus accumbens core dopamine lesions.

All rats chose the high effort/HR option on over 75% of trials pre-operatively (Fig.2a, Block A). There was no main effect of group (*F*_4,22_ = 0.388, *P* = 0.815), indicating no difference between the groups. Following surgery, however, all four lesioned groups changed their choice behaviour dramatically and made significantly fewer high effort/HR choices (on average < 20% HR arm choices at the start of Block B), whereas the shams continued to select the HR option on over 50% of trials from the beginning of Block B (mean – 72.3%; Fig.2a, Block B). Analysis of Block B using a repeated-measures anova with a within-subjects factor of day (six levels) and a between-subjects factor of group (five levels) was conducted. There was a significant overall main effect of group (*F*_4,22_ = 3.952, *P* = 0.014). There was also a main effect of day (*F*_5,110_ = 6.698, *P* = 0.001), but the group by day interaction was not significant (*F*_20,110_ = 0.793, *P* = 0.638). Duncan's pairwise comparisons showed that there was a significant difference between each of the lesion groups and the shams in Block B (all *P <* 0.05), but not between any of the lesion groups (all *P* > 0.05).

When the required effort in both the HR and LR arms was equated by placing an equivalent barrier in both arms (Block C), all rats had returned to selecting the HR option on over 68% of trials by the third day of testing (Fig.2a, Block C). A repeated-measures anova with a within-subjects factor of day (three levels) and a between-subjects factor of group (five levels) showed that there was no main effect of group in Block C (*F*_4,22_ = 1.788, *P* = 0.167) and, although there was a main effect of day (*F*_2,44_ = 12.928, *P* < 0.001), there was no interaction between group and day (*F*_8,44_ = 1.399, *P* = 0.233). This indicates that rats in all groups were able to discriminate between different sized rewards, and were able to climb the barrier and make the effortful response.

In summary, Experiment 1 demonstrated that, contrary to expectation, not only disconnection-lesioned rats, but also the ipsilateral lesion control groups were impaired on the T-maze barrier test, suggesting that a unilateral lesion of one of the target structures affected the ability to make decisions about how much effort to exert for a reward. To investigate this possibility further, in Experiment 2 we compared the performance of rats with unilateral MFC lesions, unilateral midbrain–DA and unilateral NAc–DA lesions.

### Behavioural analysis – Experiment 2

All rats chose the high effort/HR option on over 80% of trials pre-operatively (Fig.2b, Block A). There was no main effect of group (*F*_4,22_ = 0.644, *P* = 0.593), indicating no difference between the groups. Following surgery, the unilateral midbrain–DA, unilateral NAc–DA and sham rats continued to choose the HR arm on over 50% of trials during Block B, with the exception of day 2 in the NAc–DA group, where they performed below chance at 38.6% (Fig.2b, Block B). In contrast, rats in the unilateral MFC group altered their decision-making behaviour and selected the high effort/HR arm on significantly fewer trials (mean – 30.4%). A repeated-measures anova for Block B with a within-subjects factor of day (six levels) and a between-subjects factor of group (four levels) showed a significant main effect of group (*F*_3,29_ = 3.945, *P* = 0.018). The main effect of day was not quite significant (*F*_5,145_ = 2.453, *P* = 0.057), and there was no group by day interaction (*F*_15,145_ = 1.366, *P* = 0.202). Duncan's pairwise comparisons showed that the group effect was driven by significant differences between the MFC rats and each of the other three groups (all *P* < 0.05).

When the effort (barrier size) was then equated in both the HR and LR arms (Block C), the MFC rats returned to selecting the HR option on over 85% of trials by the fifth day of testing (Fig.2b, Block C). A repeated-measures anova on Block C with a within-subjects factor of day (five levels) and a between-subjects factor of group (four levels) demonstrated that there was a significant main effect of group (*F*_3,29_ = 3.182, *P* = 0.039), but also a significant main effect of day (*F*_4,116_ = 6.108, *P* = 0.002) and a group by day interaction (*F*_12,116_ = 2.604, *P* = 0.018). Analysis of simple main effects for Block C revealed that there was a significant group difference on days 1 and 2 (*P* = 0.009 on both days), but not on days 3–5 (*P* = 0.097, *P* = 0.463 and *P* = 0.491 on days 3, 4 and 5, respectively). This confirmed that by the end of Block C the unilateral MFC-lesioned rats were performing in the same way as the shams and choosing the HR option on the majority of trials. As in Experiment 1, this demonstrates that rats in all groups were able to discriminate between different-sized rewards and to exert the effortful response (i.e. climbing a barrier).

Thus, unilateral damage to the MFC resulted in a significant decrease in the number of high effort/HR choices, consistent with altered effort-based decision-making. This is likely to account for the pattern of results observed in Experiment 1.

### Behavioural analysis – effect of lesion location on effort-based decisions

#### Effect of lesion side in MFC rats

We then conducted additional analyses to explore further the nature of the deficit in the MFC-lesioned rats. We combined data from rats in Experiments 1 and 2 that had received: (i) a unilateral MFC; or (ii) a sham lesion, in order to increase the number of rats in each group. We first confirmed that there was no statistical difference between the performance of the shams in the two experiments, using a repeated-measures anova for Block B, with a between-subjects factor of experiment (two levels) and a within-subjects factor of day (six levels). This revealed no main effect of experiment or interaction between experiment and day for sham performance (*F* < 2.1, *P* > 0.05).

**Fig 3:**
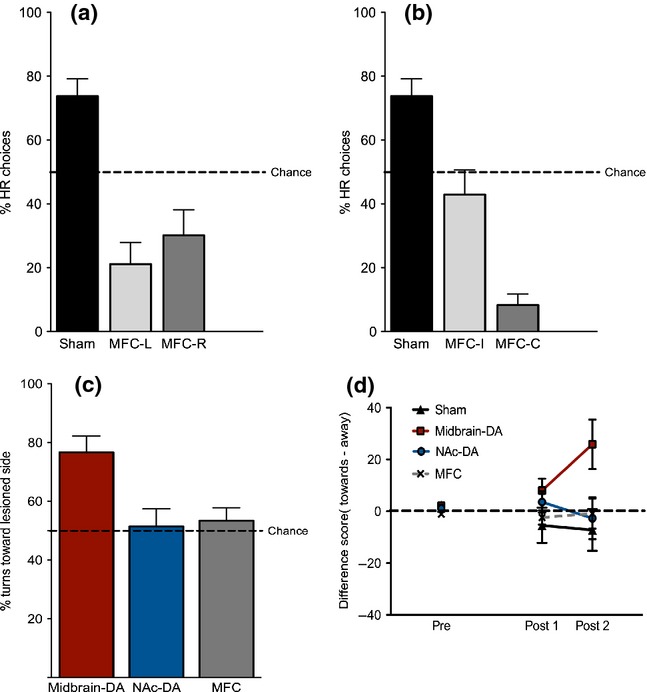
Analysis of unilateral lesion side on turning behaviour. (a) In Experiments 1 and 2, there was no difference between rats with MFC lesions in the left (MFC-L, light grey) and right (MFC-R, dark grey) hemisphere, even though both were significantly different from shams (black). (b) Analysis of sham and MFC data with respect to whether rats turned towards (MFC-I, light grey) or away from (MFC-C, dark grey) the lesioned hemisphere to obtain the high reward (HR). The MFC-C group selected the HR on significantly fewer occasions than the MFC-I group. The MFC-I group did not differ significantly from chance, while the MFC-C group did. (c) Mean number of turns towards the lesioned side made by each group on the 45° grid (chance = 50%). Midbrain-DA rats (red) made significantly more turns to ward the lesioned side than change; the other groups: NAc-DA (blue) and MFC (grey) did not. (d) Analysis of overall rotation (difference score = no. anticlockwise turns – no. clockwise turns) before (Pre) and after (Post1 and Post2) administration of AMPH. In the Post2 period, midbrain–DA rats turned significantly more often anticlockwise (towards their lesion). None of the other groups differed significantly from the shams. All error bars are standard error of the mean. Midbrain–DA, midbrain dopamine lesions; MFC, medial frontal cortex excitotoxic lesions; NAc–DA, nucleus accumbens core dopamine lesions.

We then carried out an analysis to assess whether lesion side (left – MFC-L; or right – MFC-R) differentially affected choice performance. We directly compared shams (*n* = 15), MFC-L rats (*n* = 15) and MFC-R rats (*n* = 15) by examining performance during the 6 days of post-operative testing with a 30-cm barrier in the HR arm (Block B), using a repeated-measures anova with a within-subjects factor of day (six levels) and a between-subjects factor of group (three levels). There was a highly significant main effect of group (*F*_2,42_ = 16.696, *P* < 0.001). *Post hoc* pairwise comparisons (Duncan's) confirmed that there were significant differences, both between MFC-L rats and shams (*P* < 0.05), and also between MFC-R rats and shams (*P* = 0.05), but not between MFC-L and MFC-R groups (*P* > 0.05; Fig.3a). This suggests that the hemisphere of the brain in which the MFC lesion was made did not affect the presence/absence of a deficit in choice behaviour *per se*. Both MFC-L and MFC-R groups had a strong preference for the low effort/LR arm.

#### Effect of turning direction in MFC rats

In a further analysis, we then examined whether the spatial location of the HR option in the maze relative to the lesion side affected performance levels. We grouped rats according to whether the high effort/HR arm was on the ipsilateral side of space to the lesion (MFC-I, e.g. left hemisphere lesion and left goal arm of the T-maze = high effort/HR) or the contralateral side (MFC-C, e.g. left hemisphere lesion and right goal arm of the T-maze = high effort/HR). We compared shams (*n* = 15) with MFC-I rats (*n* = 15) and MFC-C rats (*n* = 15). A repeated-measures anova for the first 6 days of post-operative testing (Block B), with a within-subjects factor of day (six levels) and a between-subjects factor of group (three levels), revealed a highly significant main effect of group (*F*_2,42_ = 31.605, *P* < 0.001; Fig.3b). *Post hoc* pairwise comparisons (Duncan's) revealed that there was a significant difference between MFC-I and shams (*P* < 0.05), and a significant difference between MFC-C and shams (*P* < 0.05). Importantly, however, there was also a significant difference between the MFC-I and MFC-C groups (*P* < 0.05), confirming that animals that had to turn away from their lesioned side to obtain the HR were much less likely to do so, and now actually exhibited a marked preference for the low effort/LR option on the effort-based T-maze task. One-sample *t*-tests on the mean scores for Block B against a test value of 50% HR choices (chance performance) showed that the MFC-I group did not significantly differ from chance (*t* = 0.914, *P* = 0.376), while the MFC-C group was significantly different from chance (*t* = 11.986, *P* < 0.001), indicating a significant preference for the low effort/LR option.

### Behavioural analysis – turning (rotational) bias

We next investigated whether the pronounced, lateralised choice behaviour of the MFC-C rats on the T-maze cost–benefit decision-making task was due to a bias caused by any unilateral motor or postural impairment. Two further tests were therefore performed with the rats from Experiment 2. These tests included animals from all groups in Experiment 2 – shams, NAc–DA, midbrain–DA and MFC rats.

#### 45° grid test

The mean percentage of turns towards the lesioned side made by each group are shown in Fig.3c. We first confirmed that the shams did not deviate from a chance value of 50% anticlockwise turns (*t* = −0.784, *P* = 0.455) and, as there is no lesion side in these animals, they were excluded from further analysis. We then analysed the percentage of turns made toward the lesioned side in each of the lesioned groups. Midbrain–DA rats showed a marked motoric turning bias, while the MFC and NAc–DA groups did not. A one-way anova showed a significant effect of group (*F*_2,23_ = 5.687, *P* = 0.011). One-group *t*-tests against a chance value of 50% showed that DA rats were significantly different from chance (*t* = 4.461, *P* = 0.003), whereas NAc–DA (*t* = 0.240, *P* = 0.818) and MFC groups were not (*t* = 0.756, *P* = 0.471). DA rats turned more often towards their lesioned side (Fig.3c).

#### Rotation after administration of AMPH

Midbrain–DA rats also had a rotational turning bias towards their lesioned side in this test, while the other groups, including the MFC rats, did not. A difference score (turns towards lesioned side minus turns away from lesioned side) was calculated for each 15-min period (Pre, Post1 and Post2; Fig.3d). A repeated-measures anova with a between-subjects factor of group (four levels: shams, midbrain–DA, NAc–DA and MFC) and a within-subjects factor of time bin (three levels) revealed that although there was no significant main effect of time bin (*F*_2,60_ = 1.630, *P* = 0.210), there was a group by time bin interaction (*F*_6,60_ = 3.404, *P* = 0.011). The overall main effect of group just failed to reach significance (*F*_3,30_ = 2.680, *P* = 0.065). A simple main effects analysis of the significant group by time bin interaction revealed that there was no effect of time bin for the shams (*F* = 1.258, *P* = 0.299), NAc–DA (*F* = 0.847, *P* = 0.439) or MFC (*F* = 1.637, *P* = 0.212) groups. However, there was a main effect of time bin for the midbrain–DA rats (*F* = 6.521, *P* = 0.005). Furthermore, there was no main effect of group in the Pre (*F*_3,30_ = 1.992, *P* = 0.136) or Post1 (0–15 min post-AMPH) time bins (*F*_3,30_ = 1.434, *P* = 0.252), but there was a significant group effect for the Post2 time bin (15–30 min post-AMPH; *F*_3,30_ = 3.610, *P* = 0.024). We therefore analysed the period 15–30 min post-AMPH (Post2) separately. One-group *t*-tests against a test value of 0 were conducted (a score of 0 would indicate equal numbers of turns in each direction, and hence no motoric turning bias). Shams, NAc–DA and MFC groups showed no significant difference from 0 (all *t* < 1.7, *P* > 0.14), but midbrain–DA rats did show a significant turning bias (*t*_8_ = 2.704, *P* = 0.027). *Post hoc* pairwise comparisons between the groups revealed a significant difference between the shams and midbrain–DA rats (*P* = 0.01), but not between any of the other pairs of groups (*P* > 0.05 in all cases).

## Discussion

In this study, we unexpectedly found that unilateral excitotoxic lesions of the MFC caused rats to alter their choice behaviour in an effort-based decision-making paradigm. Prior to surgery, all rats preferred to climb the barrier to gain the greater reward. However, rats with unilateral MFC lesions, but not with DA lesions to either the NAc (NAc–DA) or dopaminergic midbrain (midbrain–DA), changed their preference to select the low effort/LR arm. The deficit did not depend on whether the MFC lesion was in the right or left hemisphere of the brain. Strikingly, however, the deficit was particularly pronounced when the more effortful option was in the contralateral side of space to the lesion. This deficit in rats with unilateral MFC lesions represents a rare example of a selective cognitive deficit arising from a unilateral lesion in a higher-order association cortical structure, and indicates a biased lateralisation of function in the rodent MFC.

Importantly, the impairment in decision-making in the MFC rats was not due to a postural or motoric turning bias in these animals. MFC rats showed no evidence of asymmetric turning on two separate tests of rotational behaviour (a 45° grid test and the motor response to AMPH in circular bowls). In contrast, midbrain–DA rats did exhibit asymmetric rotational behaviour on both of these tests, yet their cost–benefit choice behaviour on the T-maze decision-making task was unaffected. These data thus demonstrate a double dissociation between the effects of unilateral MFC and unilateral midbrain–DA lesions in terms of turning as defined by arm choices on the effort-based decision-making T-maze task and motoric turning during two separate tests of rotational behaviour.

It is likely that these effects on rotational/motoric behaviours reflect the unintended spread of the DA depletions to the nigrostriatal dopaminergic system rather than as a consequence of DA depletion in the mesolimbic system (Ungerstedt & Arbuthnott, 1970; Steiner *et al*., 1988; see histology in Fig.1b). Notably, rats with unilateral DA depletions targeted to the NAc were neither impaired at cost–benefit decision-making nor on tests of motoric rotational behaviour, confirming that selective unilateral disruption of the mesolimbic dopaminergic system was not sufficient to cause either effect. However, it is worth pointing out that the performance (% HR arm choices) of the unilateral NAc–DA group was lower than that of the controls, and comparable to that of the unilateral MFC rats, on day 2 of Block B, although these rats quickly returned to control levels of performance during the rest of Block B. It is also worth noting that, although we did not measure the extent of the DA depletion in the current groups of rats, results from a separate experiment conducted in this laboratory and performed by several of the authors on this manuscript using identical injection co-ordinates, volumes and protocols revealed an 89% depletion of tissue DA levels in the NAc when using this procedure, as measured by high-performance liquid chromatography and electrochemical detection (Walton *et al*., 2009). Nevertheless, we cannot completely exclude the possibility that a larger unilateral DA depletion could have produced some effect on the effort-based decision-making task.

Our initial aim had been to examine the effect on effort-based decision-making of disconnecting the MFC from the mesolimbic DA system by lesioning the medial midbrain dopaminergic cells of the VTA or by selectively targeting the DA projection to the NAc (Experiment 1). Surprisingly, however, both rats with disconnection lesions (midbrain–DA–X and NAc–DA–X) *and* with ipsilateral control lesions (midbrain–DA–I and NAc–DA–I) were impaired on the effort-based decision-making T-maze task, with all lesioned animals selecting the low effort/LR option significantly more often than sham operated controls (Fig.2a). In a second experiment, it was found that only the rats with unilateral excitotoxic MFC lesions, and not those with unilateral midbrain–DA or NAc–DA lesions, were impaired at selecting the high effort/HR option. This indicates that it was the unilateral MFC lesion, which was common to all groups in Experiment 1, that contributes strongly, if not entirely, to the deficit in selecting the high effort/HR option in our first experiment.

Although rats with unilateral MFC lesions in both experiments showed a deficit in effort-based decision-making (Block B), they nevertheless all returned to choosing the HR option when an equivalent barrier was placed in both the HR and LR goal arms, meaning that they had to expend the same amount of effort to gain either reward size (Block C). This implies that the original deficit did not reflect an inability to discriminate between the HR and LR arms or an inability to climb barriers *per se*, and that the deficit was not due to gross spatial neglect. Importantly, we have shown previously that rats with bilateral anterior cingulate cortex lesions (Walton *et al*., 2003; Rudebeck *et al*., 2006), and rats with bilateral MFC lesions (derived using the same lesion coordinates as used for the unilateral MFC lesions in the present study; Walton *et al*., 2002) also perform exceptionally well on the double-barrier condition using two 30-cm barriers, choosing the HR arm on the vast majority of trials. Thus, we have shown repeatedly in bilateral lesioned animals that the impairment on this task is not due to a deficit in discriminating between reward sizes, associating those rewards with the different arms of the maze, or due to an inability to climb a 30-cm barrier. Furthermore, we have also shown that bilateral MFC-lesioned rats go back to choosing the low effort/LR arm when the barrier in the LR arm is subsequently removed, ‘after’ testing on the double-barrier condition (Walton *et al*., 2002). Taken together, these results are consistent with the hypothesis that the MFC makes an important contribution to effort-based decision-making. Notably, while all groups that included a unilateral MFC lesion were impaired at overcoming a cost to gain a greater reward, this effect was particularly pronounced when the HR option was in the hemisphere contralateral to the lesion. This raises the question of what particular role MFC plays in this type of cost–benefit decision-making task. Our results suggest that the MFC may play a crucial role in representing value in different parts of space.

The results in the current study may be explained by the MFC in each hemisphere being biased towards encoding the future benefit of a response to the contralateral side of space. Essentially, the deficit seen after the unilateral MFC lesion in the current study represents a cognitive neglect of the HR option when the effort to be overcome to obtain the reward is in the contralateral side of space to the lesion. Consistent with this, a previous study has reported lateralised, response-related deficits following unilateral damage to the MFC in rats, though not in a value-guided decision-making task. In a task requiring a sustained response to one location and then a rapid switch to a location in a different part of space, rats with lesions of MFC showed a response bias on the contralateral side to their lesion in terms of visual reaction times (Brasted *et al*., 2000). Furthermore, there are also findings from studies in humans that indicate that hand-specific values may be represented separately in the contralateral hemispheres (Gershman *et al*., 2009; Palminteri *et al*., 2009). Behaviourally, it has been shown that subliminal cues presented to either the left or right hemisphere only influence the amount of effort expended in the hand controlled by that hemisphere (Schmidt *et al*., 2010). Moreover, the expected value associated with a particular spatial response (a left hand response for stimuli presented to the left of fixation or a right hand response for stimuli to the right of fixation) but not the executed response itself, was lateralised to the contralateral MFC (Palminteri *et al*., 2009). In monkeys too, MFC (both anterior cingulate cortex and adjacent ventromedial prefrontal cortex) contains neurons that represent integrated information about the value of a target and its location in contralateral space in an attention-shifting task (Kaping *et al*., 2011).

In the present T-maze cost–benefit task, to choose to obtain the HR by climbing the barrier, the rats have first to overcome a Pavlovian bias to approach the freely available LR option in the unoccupied arm of the T-maze. Cavanagh *et al*. (2013) showed with event-related potential recordings in humans performing a go/no-go task that required them to overcome a Pavlovian response, that mid-frontal theta power correlated with successfully overcoming the Pavlovian bias. They hypothesised that this signal, which is presumed to originate in the mid-cingulate cortex (Van Veen & Carter, 2002), provides a mechanism by which striatal signals that drive Pavlovian approach behaviour could be temporarily overcome. If a unilateral MFC lesion reduces the ability to inhibit such Pavlovian responses, then the rats would show increased selection of the LR option. Such a pattern of choices would be exacerbated if there is also a degraded representation of the future reward on the other side of the barrier in the HR arm when it is in the contralesional side of space, resulting in the pronounced bias in low effort/LR options.

Notably, the robust effect of the unilateral MFC lesion in our study is in contrast to the result reported by Hauber & Sommer (2009). They found that unilateral lesions restricted to the anterior cingulate cortex, combined with ipsilateral lesions of NAc, did not impair rats' performance on a comparable T-maze effort-based decision-making task (although crucially contralateral NAc–anterior cingulate cortex lesions did result in a deficit, consistent with the importance of connections between these structures for effort-based decision-making). It is likely that this difference between the present study and that of Hauber and Sommer is due to the more extensive unilateral lesion described in the current study, incorporating the whole of MFC (anterior cingulate, prelimbic and infralimbic cortex). While we have previously shown that the anterior cingulate cortex is the critical region to allow animals to overcome effort to gain reward, it is noticeable that extensive bilateral lesions of the whole MFC cause a greater and more long-lasting change in choice behaviour on the same task than bilateral lesions restricted to the anterior cingulate cortex (Walton *et al*., 2002, 2003; Rudebeck *et al*., 2006). Importantly, this may not simply reflect lesion size *per se*, but instead may reflect a contribution from other frontal regions included in the larger MFC lesion. Indeed, it seems likely that more than one MFC subregion can contribute to this effect.

For example, although bilateral prelimbic/infralimbic cortex damage is not sufficient alone to impair effort-related decisions (Walton *et al*., 2003), it is widely believed that this region plays an important role in reward-guided behaviour. While homologies between rodents and primates are still controversial, several lines of evidence suggest that the region of the human brain that may encode lateralised spatial value-based representations (ventromedial prefrontal cortex) shares similarities with rat prelimbic cortex (Wise, 2008; Balleine & O'Doherty, 2010). Prelimbic cortex receives stronger input from the hippocampal CA1 subfield and subiculum than the cingulate cortex (Jay & Witter, 1991; Conde *et al*., 1995; Hoover & Vertes, 2007, 2011), allowing information about potential spatial goals to be relayed to MFC. Prelimbic cortex also plays an important role in control of action through interactions with motor cortex (Narayanan & Laubach, 2006). Thus, the prelimbic cortex and anterior cingulate cortex in each hemisphere may work together to allow optimal decisions to be made when options in different spatial locations differ in their relative costs and benefits.

In summary, there was a clear double dissociation between the effects of MFC lesions and dopaminergic midbrain lesions in terms of choices on an effort-based decision-making T-maze task, particularly when the HR was in the contralesional side of space, and motoric turning during two separate tests of rotational behaviour. This change in effort-based decision-making is a rare instance in which a cognitive deficit arises in rats following a unilateral lesion. We suggest that this likely reflects the strong role that the MFC plays in the integration of spatial and value information.

## Abbreviations

6-OHDA: 6-hydroxydopamine
AMPH: d-amphetamine
DA: dopamine
HR: high reward
ITI: inter-trial interval
LR: low reward
MFC: medial frontal cortex
NAc: nucleus accumbens core
PBS: phosphate-buffered saline
TH: tyrosine hydroxylase
VTA: ventral tegmental area
